# Prenatal alcohol exposure affects placental degradation—a retrospective fetal MRI study

**DOI:** 10.1007/s00330-026-12597-2

**Published:** 2026-05-06

**Authors:** Marlene Stuempflen, Michael Weber, Patric Kienast, Victor U. Schmidbauer, Sarah Glatter, Mariana C. Diogo, Rebecca Mittermaier, Felix R. M. Koenig, Tim Dorittke, Julia Binder, Gregor Kasprian, Daniela Prayer

**Affiliations:** 1 Department of Biomedical Imaging and Image-guided Therapy, Medical University of Vienna, Vienna, Austria; 2 Department of Pediatrics and Adolescent Medicine, Comprehensive Center for Pediatrics, Medical University of Vienna, Vienna, Austria; 3 Department of Neuroradiology, Sahlgrenska University Hospital, Gothenburg, Sweden; 4 Department of Radiology, University of Gothenburg, Gothenburg, Sweden; 5 Department of Obstetrics and Feto-maternal Medicine, Medical University of Vienna, Vienna, Austria

**Keywords:** Fetal alcohol spectrum disorders, Fetal MRI, Placenta, Prenatal alcohol exposure, Prenatal diagnosis

## Abstract

**Objectives:**

To provide initial insights into structural placental anomalies following prenatal alcohol exposure (PAE) utilizing routine human fetal MRI.

**Materials and methods:**

This retrospective study investigated 29 fetuses (mean gestational age 26.1 weeks, SD 3.7 weeks) subjected to PAE (PAE+) without confounding comorbidities and 29 age-matched controls without PAE (PAE−). Assessment of the placenta was performed using 1.5- and 3-T fetal MRI scans and included analysis of twelve structural parameters.

**Results:**

This study provides the first data on in vivo fetal MRI results regarding the effects of PAE on placenta structure. We identified an association of PAE with increased occurrence rates of placental lobulation, venous congestion, any type of placental hematoma, subamniotic hematoma on the placental surface, specifically, and hypercoiled umbilical cord. A trend was observed with increased placental lobulation occurring at earlier gestational ages in PAE+ patients. Overall, more structural placental anomalies were detected in women with PAE compared to unexposed controls, despite a relatively low amount of alcohol consumption (1–3 drinks per week) within the exposed group.

**Conclusion:**

Early detection of PAE is essential, considering the potentially detrimental effects of ethanol on placental structure. Fetal MRI provides a complementary tool for ultrasound-based assessment of fetus and placenta, not only in cases with congenital disorders, but also to assess effects of epigenetic factors such as PAE.

**Key Points:**

***Question*** *To assess the presence of structural placental anomalies in patients with PAE and unexposed controls in fetal MRI*.

***Findings*** *PAE is associated with increased occurrence rates of placental abnormalities - regarding the total number and type of structural anomalies*.

***Clinical relevance*** *Fetal MRI provides a complementary tool to ultrasound to assess the placenta. Early detection of structural placental anomalies, which were found to occur more frequently in alcohol exposed fetuses, may aid counseling physicians in choosing appropriate pregnancy management plans*.

**Graphical Abstract:**

**Figure:**
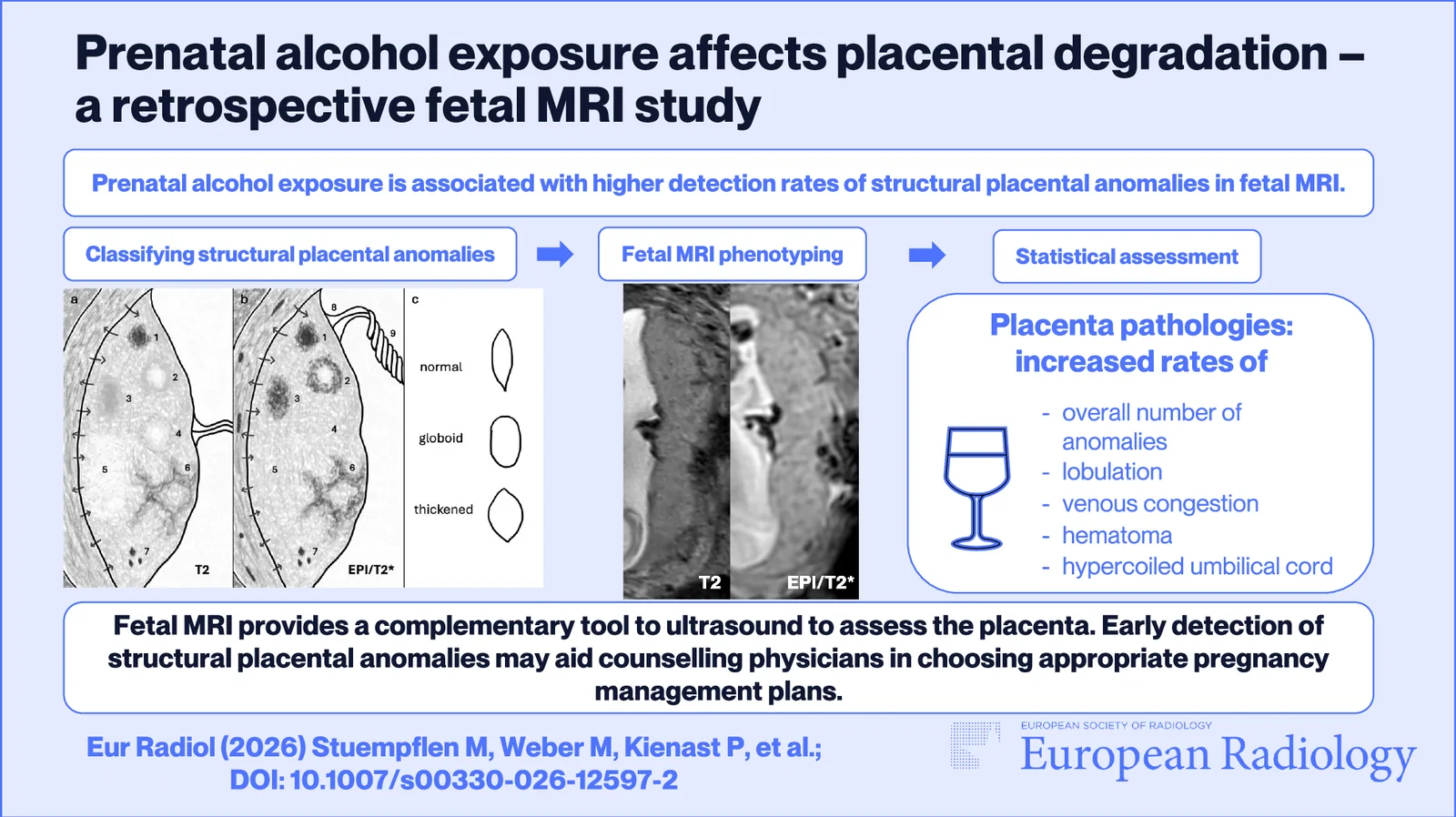

## Introduction

Prenatal alcohol exposure (PAE) can significantly alter fetal development by causing a range of structural and functional deficits affecting various organ systems, summarized as Fetal Alcohol Spectrum Disorders (FASD) [1, 2]. FASD has a prevalence of 8/1000 in the general population [3]. While 9.8% of women consume alcohol during pregnancy, 1/13 of women with PAE are expected to deliver a child with FASD [3, 4].

Alcohol-dependent changes to redox potential, nitric oxide metabolism, and hormonal imbalances have a profound effect on placental integrity [5–7]. PAE has been linked to structural and functional placental anomalies, including decreased placental size, impaired blood flow and nutrient transport, placental dysfunction, and consecutive endocrine changes within the fetus, increasing the risk for spontaneous abortion, stillbirth, preterm delivery, and low birthweight [5, 8]. While PAE may induce abnormal placental homeostasis and frequently results in premature placental ageing [5–8], we hypothesize that structural anomalies may also exacerbate the toxic effects of ethanol exposure.

Numerous molecular and histological studies have documented placental involvement in ethanol-related disorders, however, to our knowledge, no previous data are available on PAE-associated placental findings in human fetal MRI. While ultrasound is the standard imaging-modality in prenatal diagnostics, MRI provides a three-dimensional depiction of the placenta (irrespective of maternal body composition and placental location) and advanced tissue contrast (e.g., detection of placental invasion of the uterus and other structural anomalies). Available imaging-based data on placental involvement in FASD is scarce: Most studies utilized ultrasound-based Doppler measurements and detected reduced umbilical cord, placental, and embryonic blood flow measurements following maternal alcohol consumption in rodents [9–12]. Data from previous studies assessing MRI findings of placental involvement in FASD is only available in primates: Dynamic contrast-enhanced MRI and blood oxygen level-dependent MRI have been applied in rhesus macaques to quantify maternal placental perfusion and placental blood oxygenation in alcohol exposed subjects [9, 10]. These results were correlated with a reduction in fetal biometry and weight and histological signs of placental postischemic injury and microscopic infarctions [9, 10].

The aim of this study was to assess the placenta in alcohol exposed patients and to identify specific findings of ethanol-induced changes in the placenta based on imaging data of routine in vivo human fetal MRI.

## Materials and methods

### Subjects

The conduction of this study was in accordance with the Declaration of Helsinki and followed prior approval of the institutional ethics committee (approval number 2199/2017) [13]. Informed consent was acquired prior to the conduct of fetal MRI. Patients investigated in this study were initially recruited as part of a prospective study conducted between November 2018 and August 2021 [14]. The patient population of 500 pregnant women undergoing fetal MRI at a tertiary center was retrospectively reassessed during the conduction of this analysis, and subgroups of interest were selected. Following initial recruitment, maternal alcohol consumption was quantified utilizing a standardized questionnaire including parts of the PRAMS [15] and the TACE [16] assessment, which were reported to have a high sensitivity for detecting gestational alcohol intake (sensitivity 69%, positive predictive value 23% [16]). Patient data, including gestational age (as determined by ultrasound and given in gestational weeks (GWs) and days post menstruation) and maternal medical history, were obtained from institutional patient records. Patients with incomplete data were excluded from further analysis.

Patients were selected for placental assessment based on inclusion and exclusion criteria: Fetuses exposed to any amount of alcohol intake greater than zero were allocated to the patients with prenatal alcohol exposure (PAE+) group. Patients with no alcohol exposure during the entire course of the pregnancy were allocated to the patients without prenatal alcohol exposure (PAE−) group. Fetuses were analyzed if gestational age had been determined by first-trimester ultrasound, an ultrasound-based anomaly scan had been conducted, and high-quality imaging of the entire placenta was available. Patients were excluded if they presented with the following anomalies: cardiac defects, FGR (fetal growth restriction), genetic syndromes affecting the placenta, confirmed fetal infection, or intestinal disruption/abdominal wall defects. While PAE is presumed to be one cause of FGR, patients with FGR were excluded from both groups. This is due to the in vivo and imaging-based nature of this study – no additional histological and molecular testing was available to confirm whether FGR was due to PAE rather than other, potentially unknown causes (e.g., maternal coagulopathy, vascular disease). Consequently, to avoid the introduction of selection bias, all cases with FGR were excluded from further analysis.

Furthermore, patients with incomplete depiction of the placenta or the umbilical insertion at a perpendicular angle to the placenta in at least one of the required sequences (i.e., T2-weighted, EPI/T2*-, and T1-weighted imaging) were also excluded. Each alcohol exposed fetus was matched with an unexposed fetus in a 1:1 ratio based on gestational age. The MR scanner field strength was matched during case selection to allow for even distribution of 1.5- and 3.0-T-based examinations between both groups.

### Fetal MRI

Fetal MRI scans were performed on 1.5 T (Philips Ingenia/Intera) and 3 T scanners (Philips Achieva). All investigated examinations followed clinically indicated referral by a prenatal medicine expert (medical doctor with a license in obstetrics/gynecology and maternal-fetal medicine specialist) and were performed within 45 min in a supine or left recumbent position. Neither sedation nor contrast agent was applied. Scans were performed in accordance with the International Society of Ultrasound in Obstetrics & Gynecology Practice Guidelines of fetal MRI [17]. All scans included assessment of the entire fetal head and body to allow for the detection of potentially confounding comorbidities. Placental assessment required depiction of the entire placenta in at least T2-weighted (slice thickness = 3–4 mm, echo time = 100 ms, field of view = 230 mm, in-plane resolution 0.72 × 0.72 × 4 mm), Echo-Planar Imaging (EPI)/T2*-weighted (slice thickness = 3 mm, echo time = 75 ms, field of view = 300 mm, in-plane resolution 0.78 × 0.78 × 3 mm) and T1-weighted sequences (slice thickness = 4 mm, echo time = 4.6 ms, field of view = 327 mm, in-plane resolution 1.14 × 1.14 × 4 mm) and accurate delineation of the insertion of the umbilical cord. The umbilical cord had to be visualized at a perpendicular angle at the insertion site of the placenta to allow for accurate thickness measurements of the placenta.

### Placental assessment

**Fig. 1:**
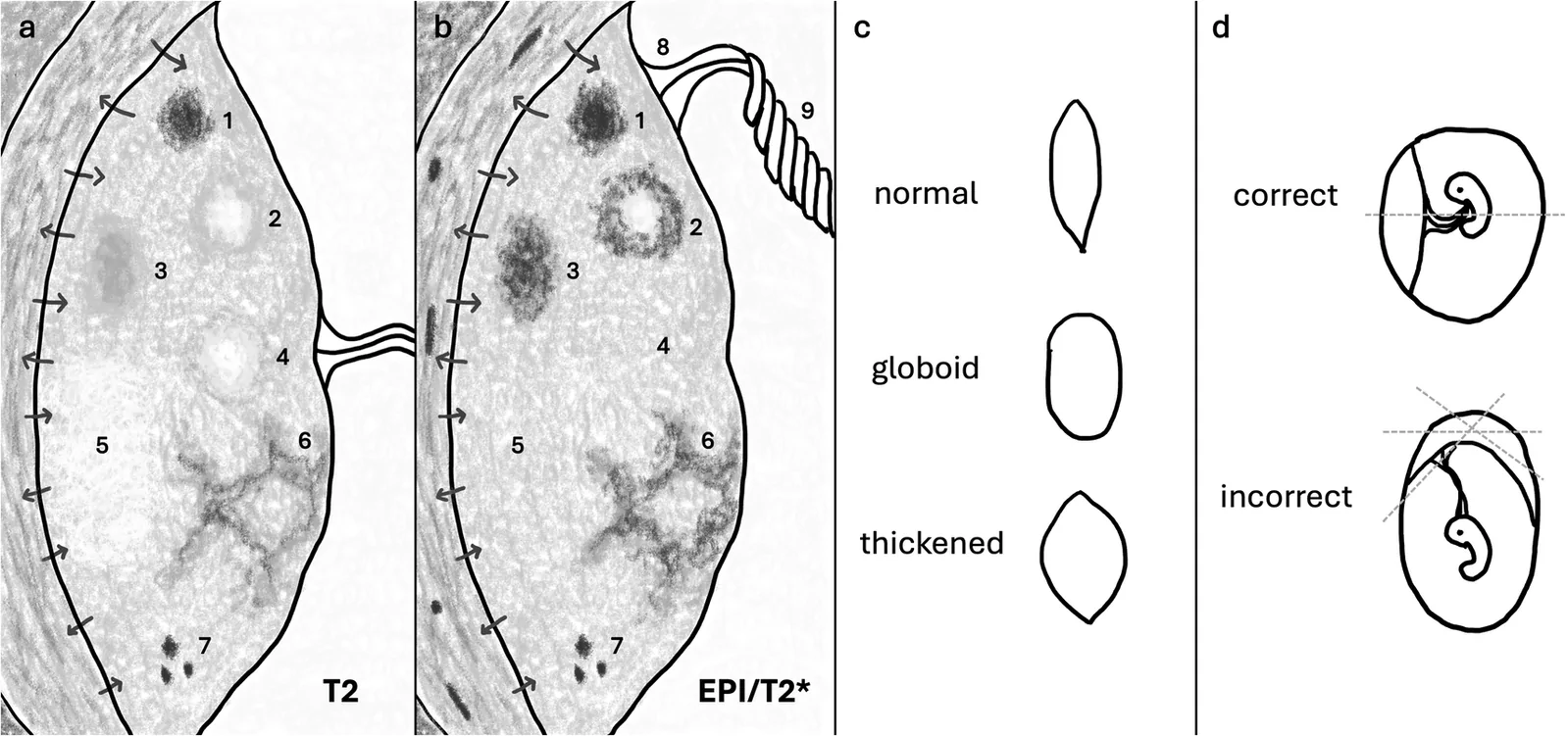
Schematic overview of assessed structural anomalies within the placenta. Placental anomalies in T2-weighted (**a**) and EPI/T2*-weighted MRI (**b**): 1—hematoma, 2—infarction, 3—thrombosis, 4—lobulation, 5—edema, 6—congestion, 7—calcifications, 8—eccentric umbilical cord insertion, 9—hypercoiled umbilical cord. **c** Normal (top), globoid (middle), and thickened placental shape (bottom). **d** Correct measurement of placental thickness and lobulation perpendicular to the insertion site of the umbilical cord (top) and exemplary incorrect measurements (bottom)

Structural assessment of the placenta was based on T2-, EPI-/T2*-, and T1-weighted imaging and involved analysis of twelve parameters: placental hypoplastic appearance, globoid shape, edema, hematoma, thrombosis, infarction, lobulation, venous congestion, thickness (measured at the umbilical cord insertion), calcifications, excentric umbilical cord insertion, and hypercoiled umbilical cord. A schematic overview of assessed structural anomalies is provided in Fig. 1. Table 1 summarizes the definitions and visual description for each parameter. Two-dimensional placenta thickness measurements were performed at the umbilical insertion site of the placenta at a perpendicular angle to the umbilical cord (Fig. 1). Two raters (20+ and 5+ years of experience in fetal MRI, respectively) conducted a structural assessment of the placenta for both groups, and disagreement between both raters was solved by consensus.

**Table 1:** Structural assessment parameters in MRI-based placental analysis

Parameter	Definition/visual description
Calcifications	T2-weighted hypointense/T1-weighted hyperintense structure without space-occupying effect
Edema	T2-weighted hyperintensity compared to age-matched normal +/− increased thickness
Globoid shape	Abnormal, globoid shape of the placenta, rather than its usual discoid shape
Hematoma	Regions of T2-/T2*-weighted hypointensity in any placental compartment +/− corresponding T1-weighted hyperintensity
Hypercoiled umbilical cord	More than three coils per 10 cm of umbilical cord
Hypoplastic appearance	Subjective visual reduction of placental volume
Infarction	Acute ischemic infarction: diffusion-restricted region Chronic ischemic infarction: delineable T2-weighted hyperintense region with hemorrhagic rim on the T2*-sequence
Lobulation	T2-weighted hyperintense regions of rounded shape with clear delineation at the placenta/myometrium interface without correlate in T2*-weighted imaging
Thickness	Defined as normal if below 60 mm, measurements performed at the insertion of the umbilical cord
Thrombosis	Intervillous thrombi: hematoma in the intervillous space Subchorionic thrombi: hematoma in the subchorionic space
Venous congestion	Pooling of blood flow in the placental veins due to restricted blood flow or increased pressure within the placenta Diffuse T2-/T2*-weighted hypointense region not restructured to an anatomical structure of the placenta

### Statistical analysis

Statistical analysis of demographic patient data and quantification of maternal alcohol consumption was conducted by applying descriptive statistics, including calculation of frequencies (provided in percentages, means, SD, and ranges).

During analysis of placental anomalies, McNemar tests were used to compare the occurrence between PAE+ and PAE− fetuses. The number of placental anomalies was compared using a paired Student’s *t*-test. Additionally, the association between the number of structural defects and gestational age was assessed using Pearson correlations for each group separately. Lobulation of the placenta was quantified, and the effect of gestational age and the number of lobules was assessed with a Spearman rank correlation calculation. *p*-values used for significance were defined as *p* < 0.05. Interrater correlation was calculated as the percentage of matching assessment parameters between both raters.

All calculations were performed using IBM SPSS Statistics for Windows, Version 28.

## Results

### Study population

**Fig. 2:**
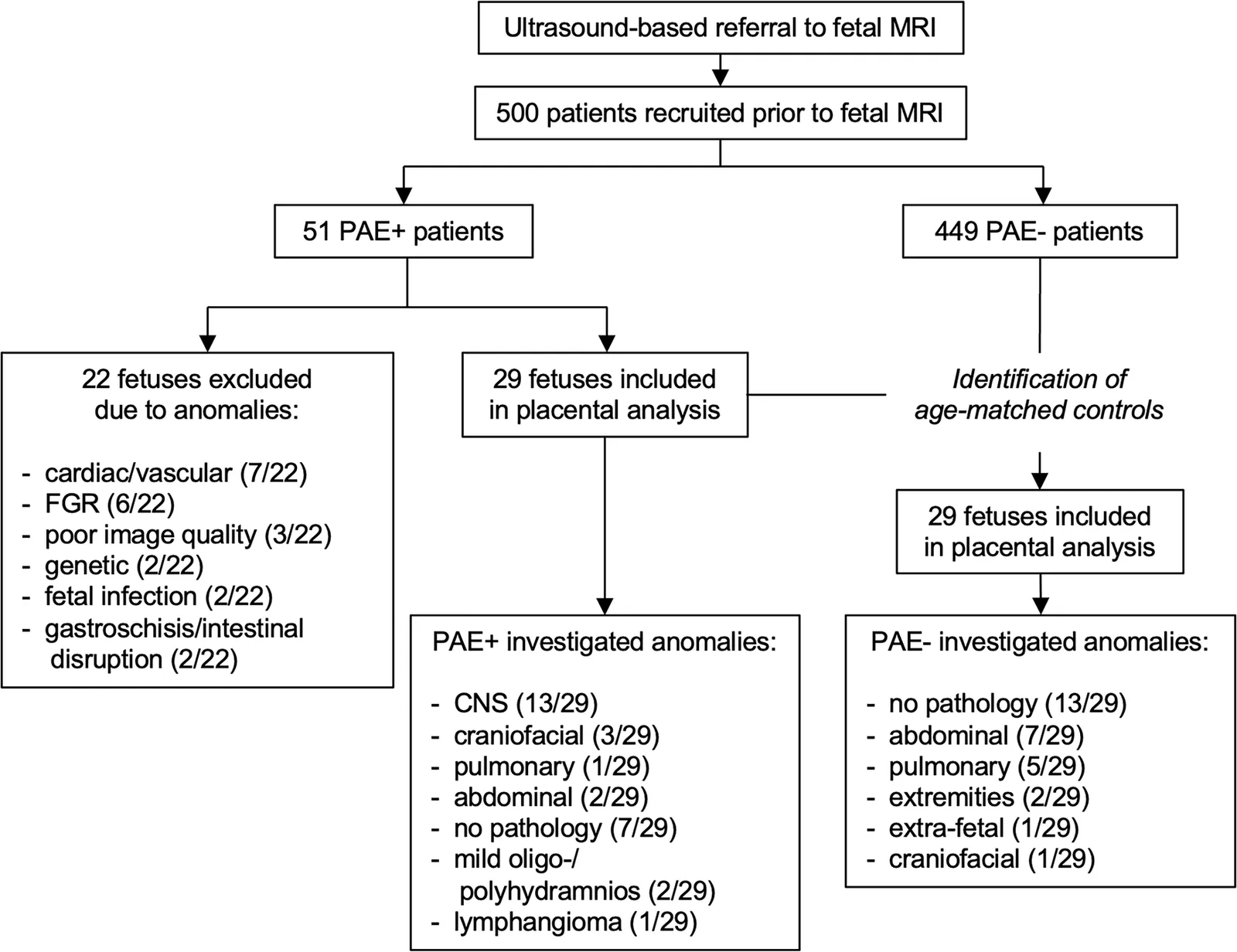
Flowchart depicting the patient recruitment and selection process. PAE+, alcohol exposed group; PAE−, non-exposed group

Among the entire patient collective of 500 pregnant women, 51 patients were identified to have consumed alcohol throughout the course of their pregnancy. Following application of inclusion and exclusion criteria, a total of 22 alcohol exposed patients had to be excluded for the following reasons: presence of confounding comorbidities including cardiac defects (*n* = 7), FGR (*n* = 6), incomplete depiction of the entire placenta or umbilical insertion site in at least one of the required sequences (*n* = 3), genetic syndromes affecting the placenta (*n* = 2), confirmed fetal infection (*n* = 2), intestinal disruption/abdominal wall defects (*n* = 2). None of the investigated patients was found to have placenta accreta. Consequently, 29 PAE+ fetuses were analyzed and compared to 29 eligible, age-matched PAE− cases selected from the existing patient collective. The patient selection process is summarized in Fig. 2, and demographic patient data is provided in Table 2.

**Table 2:** Demographic analysis of alcohol exposed (PAE+) and unexposed (PAE−) fetuses selected for placental assessment

Subjects	PAE +	PAE−
Sample size	29	29
Singleton vs twin pregnancies	28 vs 1	29 vs 0
Gestational age (GW)	26.13 ± 3.73 (20.29–33.24)	25.62 ± 3.79 (20.29–33.24)
Maternal age (years)	33.0 ± 5.15 (19–41)	33.5 ± 5.41 (24–45)
Fetal sex (male)	70%	65.5%

Sample size given in number of MRI scans. Ages given in mean ± standard deviation (range)

*GW* gestational weeks

Mean gestational ages were similar between both groups (PAE+ mean 26.13 GWs, standard deviation (SD) 3.73, and PAE− mean 25.62 GW, SD 3.79). Overall, the investigated gestational ages ranged from 20 to 33 GW and varied among PAE+ and their respective age-matched PAE− controls, with a maximum age difference of four GD. Maternal ages were comparable between both groups (PAE+ mean 33.0 years, SD 5.15, and PAE− mean 33.5 years, SD 5.41). Both groups included mostly male fetuses (PAE+ 70%, PAE− 65.5%). All but one PAE+ patient were carrying singleton pregnancies. The included twin pregnancy was diamniotic, dichorionic—from this patient, one placenta was not assessable due to incomplete imaging, thus, only one placenta from this patient was included in this assessment and matched with an unexposed control.

On average, each fetus in the PAE+ group was exposed to 1–3 standard alcoholic beverages each GW and one binge drinking event of four or more drinks on one occasion during pregnancy. According to the U.S. National Institute on Alcohol Abuse and Alcoholism, one standard drink is defined to contain roughly 14 grams of pure alcohol [18]. Fetuses allocated to the PAE− group were exposed to neither regular nor occasional alcohol consumption during pregnancy. The precise evaluation of the timing of fetal alcohol exposure was not possible due to a high degree of recall uncertainty among the investigated patients.

A summary of all structural anomalies observed in investigated patients, including ultrasound-based referral diagnoses and MRI findings is provided as Supplementary Table 1.

### Placental assessment

The interrater agreement of structural placental assessment between both reviewers was 95.1%, differences were solved by consensus as described in the methods and materials section. Patients with PAE were found to show a higher occurrence rate of structural placental changes in exposed patients compared to unexposed (*p* < 0.001). No statistically significant difference was identified regarding the effect of gestational age (PAE+ *p* = 0.54, PAE− *p* = 0.12). However, a trend was observed in both groups with increasing gestational age being associated with a higher number of structural anomalies within the placenta (PAE+ Pearson correlation coefficient = 0.12, PAE− Pearson correlation coefficient = 0.30). Particularly, patients with PAE were more likely to exhibit any type of placental hematoma (*p* = 0.013), subamniotic hematoma on the placental surface (*p* = 0.003), hypercoiled umbilical cord (*p* = 0.021), placental lobulation (*p* = 0.041), or any type of placental venous congestion (*p* = 0.012). No statistically significant association of PAE-status and higher disease occurrence was observed for placental edema, globoid placenta shape, placental infarction, increased placental thickness, placental calcifications, or placental thrombosis. One patient in the control group was reported to have a medically well-controlled chronic hypertension—her placenta was unremarkable on MRI at 21 + 0 GW. The detailed results of the structural assessment of the placenta are provided in Table 3.

**Table 3:** Observed findings following MRI-based placental assessment in alcohol exposed (PAE+) and unexposed (PAE−) fetuses

		PAE+	PAE−	*p*-value for PAE status
Calcifications		0 (0%)	0 (0%)	*n/a*
Edema		4 (13.8%)	0 (0%)	*n/a*
Hematoma	Overall	16 (55.2%)	5 (17.2%)	**0.013**
	Around the umbilical cord insertion	2 (6.9%)	3 (10.3%)	1
	Subamniotic on placental surface	12 (41.4%)	1 (3.4%)	**0.003**
	Intraparenchymal	4 (13.8%)	1 (3.4%)	0.375
Globoid shape		0 (0%)	1 (3.4%)	*n/a*
Infarction		11 (37.9%)	4 (13.8%)	0.092
Hypercoiled umbilical cord		15 (51.7%)	5 (17.2%)	**0.021**
Hypoplastic appearance		0 (0%)	0 (0%)	*n/a*
Increased thickness		2 (6.9%)	1 (3.4%)	1
Lobulation		19 (76%^*^, 101)	20 (68%, 68)	**0.041**
Thrombosis		11 (37.9%)	4 (13.8%)	0.092
Venous congestion	Overall	12 (41.4%)	3 (10.3%)	**0.012**
	Around the umbilical cord insertion	1 (3.4%)	0 (0%)	*n/a*
	Superficial	7 (24.1%)	2 (6.9%)	0.18
	Diffuse	4 (13.8%)	1 (3.4%)	0.25
Other findings (eccentric insertion of the umbilical cord)		1 (3.4%)	0 (0%)	*n/a*

Lobulation given in number of affected fetuses (percentage of each respective group, number of total counted lobules). Other parameters given in number of affected fetuses (percentage of each respective group). Bold values indicate statistical significance (*p* < 0.05).

*n/a* not applicable

^*^ Four cases among the PAE+ group presented with placental edema, making precise delineation of placental lobules based on MRI unreliable. Consequently, only 25 PAE+ patients were assessed regarding lobulation.

**Fig. 3:**
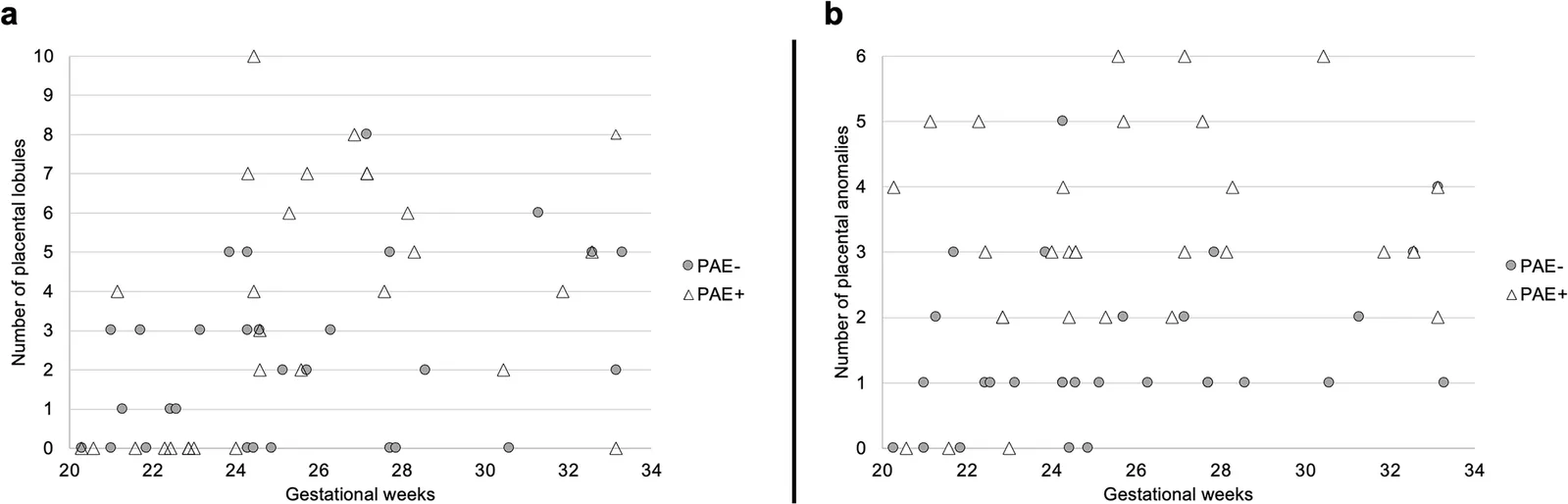
Distribution of the total number of placental anomalies (**a**) and the number of placental lobules in each patient (**b**) observed throughout gestation, depending on PAE status. Color coding: white triangles: PAE+ group, gray circles: PAE− group

**Fig. 4:**
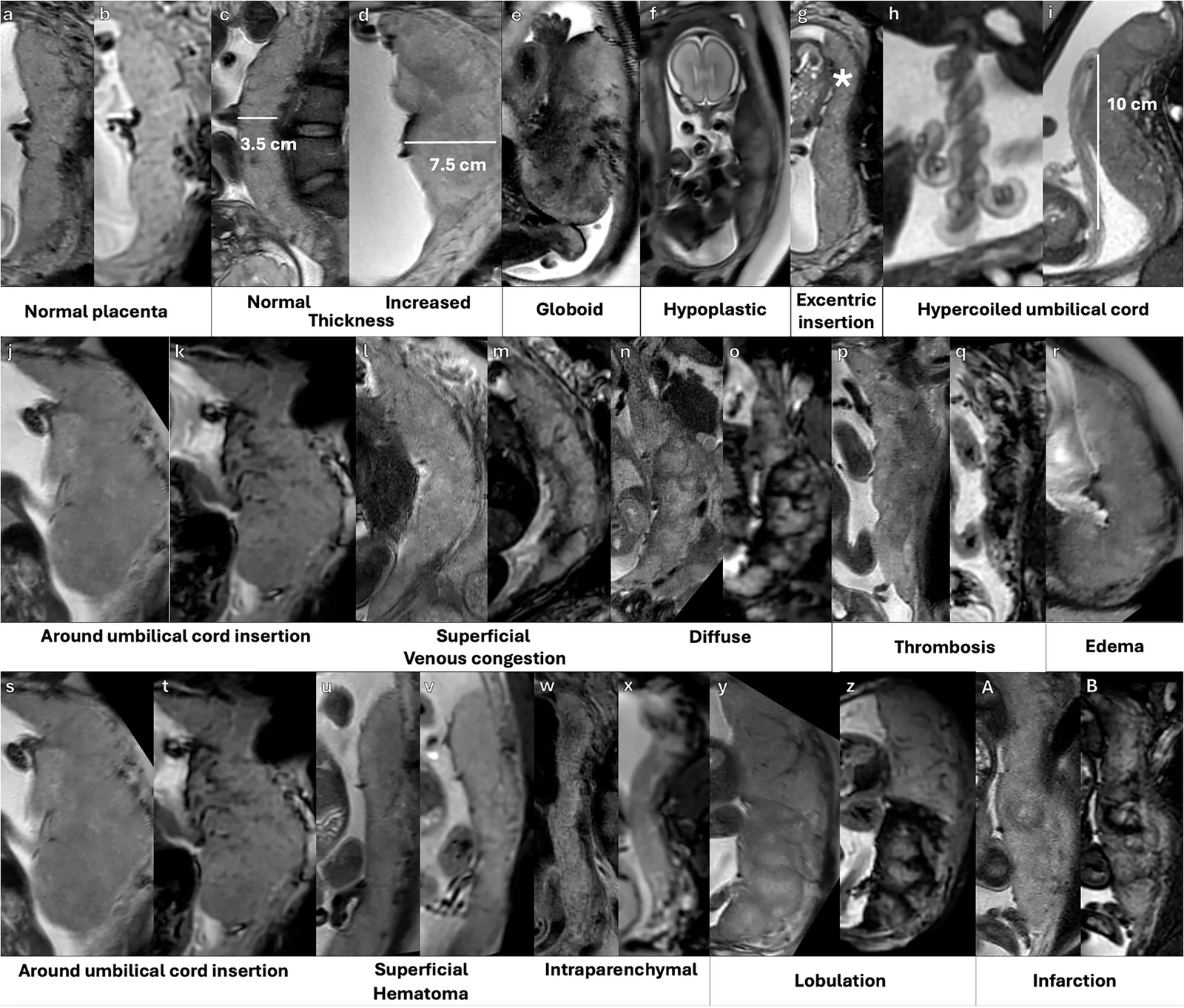
Exemplary placental anomalies depicted in T2-weighted and EPI/T2*-weighted imaging. **a**, **b** Normal placenta (21 + 2 GW). **c**, **d** Normal (**c**, 28 + 4 GW) and increased (**d**, 24 + 2 GW) placental thickness. **e** Globoid placenta shape (28 + 2 GW). **f** Hypoplastic placenta (24 + 5 GW). **g** Asterix indicating an eccentric insertion of the umbilical cord (26 + 4 GW). **h**, **i** Hypercoiled umbilical cord (25 + 4 and 20 + 1 GW). **j**–**o** Venous congestion of the placenta—around the insertion of the umbilical cord (**j**, **k**, 22 + 2 GW), in superficial placenta parts (**l**, **m**, 33 + 1 GW), and with diffuse involvement of the placenta (**n**, **o**, 25 + 2 GW). **p**, **q** Thrombosis of the placenta (24 + 0 GW). **r** Placental edema (22 + 2 GW). The presence of edema does not allow for assessment of lobulation. **s**–**x** Placental hematoma—around the insertion of the umbilical cord (**s**, **t**, 22 + 2 GW), in superficial placenta parts (**u**, **v**, 22 + 6 GW), and with diffuse involvement of the placenta (**w**, **x**, 22 + 0 GW). **y**, **z** Placental lobules (and diffuse venous congestion) in the lower placenta in twin pregnancy (24 + 3 GW). Edematous placenta of the second twin on top. A, B: Placental infarction (30 + 3 GW). GW, gestational weeks

Quantification of placental lobules was conducted within three distinct image slices: this included the image slice depicting the insertion of the umbilical cord and the two slices adjacent to it in each direction. As previously mentioned, the occurrence of placental lobulation was found to be associated with PAE-status (*p* = 0.041). While no association was found between the number of observed lobules and gestational age (PAE+: *p* = 0.068, PAE−: *p* = 0.134), an age-dependent trend was identified in both groups (PAE+: Spearman-Rho coefficient = 0.371, PAE−: Spearman-Rho coefficient = 0.285). Table 4 summarizes quantitative results regarding placental lobulation. Figure 3 visualizes the distribution of the total number of observed placental anomalies in each patient (a) and the number of observed placental lobules in each patient (b). Figure 4 provides an overview of exemplary structural anomalies within the placenta.

**Table 4:** Analysis of PAE status on detection rates of placental lobulation, including the effect of gestational age

					Range		Percentile		Effect of gestational age on number of observations	
	*n*	Mean	Median	SD	Min	Max	25th	75th	Spearman-Rho	*p*-value
PAE+	25	4	4	3	0	10	2	7	0.371	0.068
PAE−	29	2.3	2	2.2	0	8	0	3	0.285	0.134

Including only patients without placental edema, as the presence of placental edema does not allow for quantification of lobules

*n* sample size, *SD* standard deviation, *Min* minimum, *Max* maximum, *PAE*+ alcohol-exposed patients, *PAE*− unexposed patients

## Discussion

This study provides the first insights into the detrimental effects of PAE on the structural integrity of the placenta, visualized in prenatal MRI. We identified PAE to be associated with various structural placental anomalies, including increased placental lobulation, venous congestion, any type of placental hematoma, subamniotic hematoma on the placental surface, specifically, and hypercoiled umbilical cord. More structural placental anomalies were detected in women with gestational alcohol consumption compared to age-matched, unexposed controls, despite a relatively low amount of alcohol consumption within the exposed group. While physiological placental maturation is histologically characterized by increasing lobulation, particularly during the third trimester, and may, thus, to some degree be considered normal [19], we identified a trend of earlier placental lobulation in PAE+ patients.

While MRI-based data on placental lobulation are limited, increased lobulation has been linked to premature aging of the placenta and has been associated with heterogeneity in placental oxygen and nutrient transport, potentially affecting fetal nutrient supply and growth [20]. These results emphasize the importance of standardized semiquantitative assessment of the placenta in fetal MRI.

Numerous studies have investigated the histological and molecular changes within the placenta following PAE, which involve the redox potential, nitric oxide metabolism, hormonal imbalances, and abnormal fetal energy homeostasis [6, 7, 21, 22]. Co-occurrence of maternal comorbidities, including obesity, diabetes, hypertension, and preeclampsia, may exacerbate the detrimental effects of ethanol [23–27]. Furthermore, the presence of structural anomalies makes the placenta even more susceptible to the toxic attributes of alcohol. This results in higher occurrences of placental dysfunction, decreased placental size, impaired placental blood flow and nutrient transport, and consecutive endocrine changes within the fetus, increasing the risk for early pregnancy loss and fetal developmental delay [5, 6, 8]. This is highlighted by the increased FGR rates in alcohol exposed patients (11.7%) excluded from this study compared to the general prevalence (10%) [28]. However, no histo-molecular proof that alcohol was the sole cause of FGR in these patients was available.

While biomolecular effects of PAE have been studied meticulously, data on radio-morphological findings on placental involvement in FASD are limited: few studies have applied ultrasound and Doppler measurements and identified decreased fetal, placental, and umbilical cord blood flow following PAE [9–12]. MRI-based assessment of alcohol-dependent placental changes in primates identified reduced placental perfusion and lower oxygen exchange in alcohol exposed subjects, correlating with reduced fetal biometry and weight and higher rates of placental postischemic injury and microscopic infarctions [9, 10].

This study provides initial insights into ethanol-related changes in the human placenta utilizing in vivo fetal MRI: While the observed placental changes are non-specific, we identified increased susceptibility of ethanol-exposed placentas to develop structural anomalies. These findings correlate with histological and imaging-based studies conducted with other imaging modalities or in non-human subjects.

Considering the pernicious effects of placental dysfunction on fetal development, early structural assessment of the placenta and standardized screening for PAE are essential. Fetal MRI provides a complementary tool to ultrasound-based placental assessment. Structural anomalies within the placenta may result in FGR, and additional fetal anomalies may be observed. Vascular placental anomalies in fetal MRI have been identified as an additional risk factor for pregnancies at risk for adverse outcomes and fetal death, independently of umbilical artery Doppler status [29]. Consequently, the reported findings may serve as soft markers for PAE in fetal MRI and highlight that earlier detection of PAE and placental injury in fetal MRI may aid counseling physicians, as pregnancy management may profoundly change depending on the severity of detected anomalies.

### Clinical implications

This study highlights the necessity for standardized screening for PAE. While fetal MRI currently plays no role in the detection of PAE, its clinical application in patients with PAE may provide additional phenotyping potential. Patient education regarding the detrimental effects of PAE should start prior to conception, and alcohol screening should be performed at every prenatal check-up. The alarmingly low rate of only 3.9% (2/51 cases) of referring prenatal medical professionals having knowledge of their patients’ gestational alcohol consumption emphasizes the lack of proper screening.

Fetal MRI reports should include semiquantitative structural assessment of the placenta to complement findings detected in routine ultrasound. As FASD are often mis- and underdiagnosed, establishing an earlier diagnosis prenatally may improve long-term outcomes as specialized treatment can be implemented at earlier developmental stages.

### Research implications

The reported findings highlight MRIs’ ability to serve as a tool for the detection of congenital disorders, as well as the assessment of effects of epigenetic factors, including PAE. Previous studies have identified various genetic variants causing a predisposition to ethanol-related injury [30]. Future studies should aim to verify the presented results in larger patient collectives, including histological and genetic assessment and analysis of the precise duration and degree of PAE, and a prospective comparison of ultrasound and MRI findings.

### Strengths and limitations

Due to the imaging-based nature of this study, the underlying molecular mechanisms of PAE-associated anomalies could not be determined—associations with PAE were investigated rather than causality of observed findings. Furthermore, the correlation of timing and degree of PAE and severity of MRI findings was limited due to the sample size of investigated patients. Additionally, assessment of PAE-related structural anomalies of the fetus was outside the scope of this study.

PAE assessment was based on maternal self-reporting harboring an intrinsic risk for underreporting: Women reporting any alcohol consumption (even if as small as a singular alcoholic drink) were allocated to the PAE+ group, while women in the PAE− group may have inaccurately recalled a negative history of alcohol exposure. This may act as a significant confounder in group allocation. Considering this intrinsic limitation, the applied questionnaire included parts of the PRAMS and TACE assessments, which have been recommended by the American College of Obstetricians and Gynecologists [31] and were previously applied in numerous published studies investigating PAE. Furthermore, the percentage of patients reporting PAE in our collective was similar to previous larger cohorts [4].

To provide an accurate representation of patients on the FASD spectrum [32, 33], we included some patients with various structural anomalies, excluding patients with confounding comorbidities potentially affecting the placenta.

MRI-based assessment harbors intrinsic limitations in the differentiation of placental lobules in co-occurrence with edema. Furthermore, differentiation of infarction and lobulation may be challenging in MRI as infarcted areas transition into placental lobules during a gradual process of cellular restructuring and remodeling. Additionally, MRI-based differentiation of calcifications from subacute hemorrhages (i.e., methemoglobin) is challenging. Lastly, semiquantitative evaluation, including assessment of placental lobules at the level of umbilical cord insertion, might not reflect the state of the whole placenta. However, the applied methodology was selected to provide a reproducible approach to placental assessment despite these intrinsic limitations. Future studies should include histological and ultrasound correlation to verify the presented results and assess placental calcifications, as no calcifications were observed in this patient collective.

## Supplementary information

ELECTRONIC SUPPLEMENTARY MATERIAL

## Abbreviations

EPI: Echo-planar imaging
FASD: Fetal alcohol spectrum disorders
FGR: Fetal growth restriction
GW: Gestational week
PAE−: Patients without prenatal alcohol exposure
PAE: Prenatal alcohol exposure
PAE+: Patients with prenatal alcohol exposure
SD: Standard deviation
