# Supplementary material for: Prenatal alcohol exposure affects placental degradation—a retrospective fetal MRI study
Source: Eur Radiol. 2026 May 6;36(9):7118–28. doi: 10.1007/s00330-026-12597-2 (PMC13451260; doi:10.1007/s00330-026-12597-2)
Supplement: Supplementary file 1 [file 330_2026_12597_MOESM1_ESM.pdf]

Prenatal alcohol exposure affects placental degradation – a retrospective fetal MRI study

ELECTRONIC SUPPLEMENTARY MATERIAL

PAE	Sex	Reasons for referral/ suspected diagnosis	Final diagnosis following MRI
+	m	complete callosal agenesis	complete callosal agenesis, cortical heterotopia, abnormal gyration, abnormal position/configuration of the foot
+	m	syndactyly in previous pregnancy	no pathology
+	m	complete callosal agenesis	ventricular asymmetry
+	m	liver cyst	no pathology
+	f	abnormal bowels	intracerebral hemorrhage, hepatosplenomegaly, hemochromatosis
+	m	ventriculomegaly	ventriculomegaly
+	m	lymphangioma of the leg	lymphangioma of the leg
+	f	alcohol consumption during pregnancy	small cerebral & cerebellar biometry
+	m	sacrococcygeal region not visible	no pathology
+	m	partial callosal agenesis	abnormal skull shape
+	f	meningomyelocele	Chiari II malformation, myelocele
+	f/m	twins – fetus 1 oligohydramnios	twins – oligohydramnios
+	m	lissencephaly	lissencephaly
+	m	complete callosal agenesis	partial callosal agenesis
+	m	cleft lip, jaw, and palate	no pathology
+	f	unilateral kidney agenesis	unilateral dysplastic pelvic kidney
+	f	congenital diaphragmatic hernia	diaphragmatic eventration, lung hypoplasia
+	f	complete callosal agenesis	partial callosal agenesis, Probst bundles
+	m	subcapsular liver hematoma, rhesus incompatibility	signal alterations in liver, rhesus incompatibility
+	m	ventriculomegaly, abnormal gyration	asymmetrical ventriculomegaly, plexus cysts
+	m	lissencephaly in previous pregnancy	no pathology
+	m	exophthalmos	midface hypoplasia, choanal atresia, exophthalmos
+	f	no pathology	no pathology
+	f	arachnoidal cyst	arachnoidal cyst
+	m	polyhydramnios	polyhydramnios
+	m	complete callosal agenesis	complete callosal agenesis, ventriculomegaly
+	u	ventriculomegaly, megacisterna magna	Septum pellucidum agenesis, ventriculomegaly, megacisterna magna, subependymal heterotopia, suspected filamin mutation
+	m	cleft lip, jaw, and palate, hypertelorism	cleft lip, jaw, and palate, hypertelorism, prominent forehead
+	m	alcohol consumption during pregnancy	no pathology
-	m	diaphragmatic hernia	diaphragmatic eventration right

-	m	club feet	club foot
-	m	steatosis hepatis	utuerus myomatosus, no fetal pathology
-	m	heterotaxy syndrome in previous pregnancy	no pathology
-	f	club feet	club feet
**PAE**	**Sex**	**Reasons for referral/ suspected diagnosis**	**Final diagnosis following MRI**
-	f	unilateral kidney agenesis	unilateral kidney agenesis
-	f	congenital pulmonary airway malformation	congenital pulmonary airway malformation
-	m	duodenal atresia	elongated gallbladder
-	m	cisterna magna hemorrhage	no pathology
-	f	gall bladder anomaly	elongated gallbladder
-	m	tuberous sclerosis in previous pregnancy	no pathology
-	m	abdominal cyst	no pathology
-	m	congenital pulmonary airway malformation	congenital pulmonary airway malformation
-	m	dilated umbilical vein	Intraabdominal aneurysm of the umbilical vein
-	m	asymmetrical ventriculomegaly	no pathology
-	m	congenital pulmonary airway malformation, pulmonary sequestration	pulmonary sequestration
-	m	abdominal cyst	elongated gallbladder
-	f	cleft lip and jaw	cleft lip and jaw
-	f	distended bowel loop	no pathology
-	f	choledochus cyst	gallbladder agenesis
-	m	increased head size	no pathology
-	f	congenital pulmonary airway malformation	congenital diaphragmatic hernia
-	m	ventriculomegaly, macroglossia	no pathology
-	m	hyperechogenic spots in fetal liver	no pathology
-	m	partial callosal agenesis	no pathology
-	m	abdominal mass	no pathology
-	f	fetal demise in previous pregnancy	no pathology
-	m	dorsal pancreas agenesis	dorsal pancreas agenesis
-	f	bowel atresia	no pathology

Supplementary Table 1: Ultrasound-based referral diagnoses and MRI findings in fetuses selected for placental assessment

PAE – prenatal alcohol exposure, m – male, f – female, u – undifferentiable, twins indicated with "/"
